# Ustekinumab during pregnancy and lactation: drug levels in maternal serum, cord blood, breast milk, and infant serum

**DOI:** 10.1186/s40780-022-00249-8

**Published:** 2022-07-01

**Authors:** Jumpei Saito, Kayoko Kaneko, Hiroyo Kawasaki, Takeshi Hayakawa, Naho Yakuwa, Tomo Suzuki, Haruhiko Sago, Akimasa Yamatani, Atsuko Murashima

**Affiliations:** 1 Department of Pharmacy, National Center for Child Health and Development, Okura 2-10-1, Setagaya-ku, Tokyo, 157-8535 Japan; 2 Division of Maternal Medicine, Center of Maternal-Fetal, Neonatal and Reproductive Medicine, National Center for Child Health and Development, Tokyo, Japan; 3 Sangenjaya Hayakawa Clinic, Tokyo, Japan; 4 Japan Drug Information Institute in Pregnancy, National Center for Child Health and Development, Tokyo, Japan; 5 Division of Obstetrics, Center for Maternal-Fetal, Neonatal and Reproductive Medicine, National Center for Child Health and Development, Tokyo, Japan

**Keywords:** Ustekinumab, Ulcerative colitis, Pregnancy, Lactation

## Abstract

**Background:**

Patients with ulcerative colitis (UC) may be concerned about medication safety during preconception, pregnancy, and lactation, and they should be closely followed up to ensure that UC activity is controlled during the perinatal period. Reported information on the safety of ustekinumab during pregnancy and lactation is limited. In this case report, we examined the safety of ustekinumab in a fetus and breastfed infant with reference to drug concentrations in maternal serum, cord blood, breast milk, and infant serum.

**Case presentation:**

A 36-year-old female who developed hematochezia and was diagnosed with ulcerative colitis at age 24 was pregnant with her first child. During pregnancy she was treated with subcutaneous bimonthly ustekinumab, at a dose of 90 mg, until 29 weeks of gestation. Her ulcerative colitis symptoms remained in remission. At 38 weeks of gestation she underwent cesarean section and delivered a healthy female infant weighing 3043 g and with no congenital malformations. The infant received routine vaccinations with no adverse events. Ustekinumab treatment was resumed at 7 weeks postpartum.

The ustekinumab concentration in maternal serum at 12 days after injection (30.7 weeks of gestation) was 7968.5 ng/mL, and it decreased to 106.1 ng/mL at 114 days after the last dose. In cord blood, the ustekinumab concentration was 1131.2 ng/mL at 65 days after the last dose; this was 2.5 times higher than that in the maternal serum, which was consistent with a previous report. Ustekinumab was detected in infant serum collected at 71 days after the last maternal dose (299.0 ng/mL), with rapid elimination from the infant’s body. In breast milk, the maximum ustekinumab concentrations were 13.6 ng/mL at 9 days after the last maternal dose, respectively. The ratio of the calculated areas under the time-concentration curves of ustekinumab in breast milk and maternal serum was 0.0008 (257.1/327632.7), which was comparable with a previous human study.

**Conclusion:**

The placental transfer and breast milk secretion of ustekinumab in our case were comparable with previous reports. Use of ustekinumab during pregnancy and lactation was feasible in this case. Further research is needed to clarify the safety of ustekinumab during pregnancy and lactation.

## Background

Patients with ulcerative colitis (UC) may be concerned about the safety of medications before pregnancy, during pregnancy, and during lactation [1–4], and should be followed closely during the perinatal period to ensure that UC activity is under control [3]. While active UC increases the risk of adverse pregnancy events [ 5–7], disease remission has been reported to lead to improved birth rates [8, 9] and favorable pregnancy outcomes [4]. In pregnant woman with UC, 5-aminosalicylate (5-ASA), corticosteroids, and thiopurines are safe for use during pregnancy, while methotrexate and tofacitinib should only be used with extreme caution. Anti-tumor necrosis factor (TNF) agents (except certolizumab), vedolizumab, ustekinumab, and tofacitinib readily cross the placenta, and therefore may theoretically affect fetal development. Previous reviews and a multicenter prospective study indicated that ustekinumab is thought to be safe in pregnancy and lactation [10–13]. However, there is little information on the safety of ustekinumab in Japanese patients. In this case report, we examined the safety of ustekinumab in a fetus and breastfed infant with reference to drug concentrations in maternal serum, cord blood, breast milk, and infant serum.

## Case presentation

A 36-year-old female who developed hematochezia and was diagnosed with UC at age 24 was pregnant with her first child. Oral 5-ASA (Lialda® tablets 4800 mg, Mochida Pharmaceutical Co. Ltd., Tokyo, Japan) and oral corticosteroids were administered. Six months before the woman initially found out that she was pregnant for the first time (G1, P0), she developed a severe perianal abscess that was considered to represent a symptom flare, and she was treated with systemic corticosteroids. Two weeks before learning of her pregnancy, she was treated with subcutaneous ustekinumab at a dose of 90 mg every 2 to 3 months. Ustekinumab treatment was continued until 29 weeks of gestation (the second, third, and fourth treatments were administered at 5, 17, 29 weeks of gestation, respectively). The patient was also treated with 5-ASA at 4800 mg per day during pregnancy and lactation. Corticosteroids (prednisolone 5–30 mg) had been administered since the start of the UC treatment, depending on the severity of UC, but were discontinued with the start of ustekinumab. During pregnancy, UC symptoms remained in remission and no relapse of UC during treatment with ustekinumab. During ustekinumab treatment, C-reactive protein ranged from 0.02 to 0.3 mg/dL, white blood cell counts ranged from 5000 to 9000/μL, and haemoglobin was maintained above 11 g/dL.

The patient delivered a female infant weighing 3043 g by cesarean section at 38 weeks and 3 days of gestation. The infant’s Apgar scores at 1 minute and 5 minutes were 8 and 9, respectively. Due to decreased oxygen saturation (SpO2 was 75%), continuous positive airway pressure therapy with 1 L/min of oxygen was initiated within 5 minutes after birth. Oxygen treatment was discontinued within 20 minutes and the infant was admitted to the growing care unit. Her respiratory condition improved 1 day after delivery. No medication or circulatory support was needed during admission. The infant exhibited normal developmental progress, with no congenital malformations, and was discharged at 21 days of age.

The mother developed postpartum hypertension, and 10 mg amlodipine daily was initiated. Her systolic blood pressure was maintained at less than 140 mmHg. At 5 days postpartum, lower abdominal pain developed, and puerperal endometritis was suspected. Ampicillin-sulbactam was administered intravenously. An abscess was found in the vesicouterine pouch, and was drained. *Mycoplasma homisis* was detected in the abscess drainage fluid. Blood and amniotic fluid cultures were negative. An elevated serum C-reactive protein value gradually decreased after the antibiotic treatment and abscess drainage, and she was discharged at 21 days postpartum. Subcutaneous ustekinumab treatment was resumed at 7 weeks postpartum.

During a 3-month lactation period, the infant was partially breastfed, with over 50% of nutrition derived from breastfeeding. Although the mother continued taking 4800 mg of 5-ASA daily during lactation, the infant demonstrated no detectable drug-related adverse events at the 1- and 3-month postpartum health checkups. As part of the infant’s routine childhood vaccination regimen, she received the *Haemophilus influenzae* type b conjugate vaccine, hepatitis B vaccine, combination diphtheria–pertussis-tetanus–inactivated polio vaccine (DPT-IPV), and 13-pneumococcal polysaccharide vaccine (PCV13), and experienced no adverse events after any of these. In line with the guidelines for infants exposed to non-routine antibody therapy in pregnancy, live vaccines, including the rotavirus and Bacille Calmette-Guérin vaccines, were not administered until 6 months of life.

The detected ustekinumab concentrations in the maternal serum, cord blood, infant serum, and breast milk were shown in Table 1. The ustekinumab concentration in maternal serum at 12 days after the fourth subcutaneous treatment (at 30.7 weeks of gestation) was 7968.5 ng/mL, and it decreased continuously thereafter, reaching a value of 106.1 ng/mL at 114 days after the last dose (at 48 days postpartum). In cord blood, the ustekinumab concentration was 1131.2 ng/mL at 65 days after the last dose, which was 2.5 times higher than the concentration in maternal serum. The concentration in infant serum collected at 71 days after the last maternal dose (at 5 days postpartum) was 299.0 ng/mL.

**Table 1:** Ustekinumab concentrations in the maternal serum, cord blood, infant serum, and breast milk

Gestational weeks	Postpartum Days	Ustekinumab injection	Ustekinumab concentration (ng/mL)			
			Maternal serum	Cord blood	Infant serum	Breast milk
−3	− 289	90 mg subcutaneous				
5	− 233	90 mg subcutaneous				
17	− 149	90 mg subcutaneous				
29	−65	90 mg subcutaneous				
30	−54		7968.5			
35	−19		1359.7			
38	0			1131.2		
	1		459.6			
	5		426.3		299.0	
	7		395.8			
	9		373.8			
	12		343.9			
	13		342.4			
	14		306.4			
	15		291.6			
	17		280.5			
	19		234.8			
	21		223.5			
	48.2	90 mg subcutaneous				< LLOD
	48.3		106.1			
	48.5					< LLOD
	49					1.5
	57					13.6
	64					10.8
	71					7.9
	77		2834.1			
	78					3.4
	105		1383.7			

*LLOD* lower limit of detection

The ustekinumab concentration in breast milk reached a peak at approximately 9 days after the fifth treatment (at 48 days postpartum), and decreased gradually thereafter. The maximum ustekinumab concentrations in breast milk was 13.6 ng/mL at 9 days after the last maternal dose (at 57 days postpartum). The ratio of the calculated areas under the time-concentration curves (AUCs) of ustekinumab in breast milk and maternal serum, determined using the trapezoidal method, was 0.0008 (257.1/327632.7).

After the study was approved by our institution’s ethics committee and the participant provided written informed consent, ustekinumab levels in maternal serum, cord blood, breast milk, and infant serum were evaluated. Ustekinumab levels were measured using an anti-IL12/23 humanized IgG enzyme-linked immunosorbent assay (Alpha Diagnostic International, San Antonio, TX, USA).

The calculated lower limit of detection (LLOD)—determined by extrapolating the concentration at a OD450/620 nm signal equal to background plus three standard deviations of the background signal—was 1.0 ng/mL. Accuracy, was determined by repeated analysis of a positive control ustekinumab in serum and breastmilk, was within 10% for serum and breastmilk sample, respectively. Intra- and inter precision, a measure of the degree of repeatability of an assay, was within 10% for serum and breastmilk, respectively. All samples were diluted by the dilution buffer of the kit to the range of the calibration curve (1.0 to 100 ng/mL). Although ustekinumab is not stable in breast milk at room temperature [14], samples collected in this study were immediately stored at − 20 °C, and analyses were performed under cool conditions. To resolve discrepancies with other studies, it will be necessary to clarify the process of ustekinumab secretion into breast milk.

## Discussion and conclusion

We experienced a pregnant woman with UC who was treated with ustekinumab during pregnancy and lactation, including the first trimester. Ustekinumab transfer into breast milk was evaluated using the AUC. The ratio of ustekinumab in cord blood to maternal level was 2.5, which corresponded to the value in a previous report [15]. The concentration of ustekinumab in breast milk was 1/1400th of that in maternal serum, which was similar to findings in a previous study on macaques [16] and a case study in nursing mothers with Crohn’s disease [17]. However, the concentration in breast milk was lower than that in other studies, in which the ranges were 0.72–1.57 μg/mL at peak and 0.16–0.82 μg/mL at trough, respectively [14, 17, 18]. Although the dosing schedule of ustekinumab during pregnancy and lactation and the calculation method for the milk to serum may affect a calculated value, the reported milk to serum ratio was ranged from 0.0003 to 0.005 [17, 18], that was comparable with our case.

Regarding the safety of the breastfed infant, there were no negative findings except for decreased oxygen saturation at birth. Previous studies also reported no harmful effects in infants [14, 17, 18]. In this study, the ustekinumab concentration in the infant’s serum was 598.0 ng/mL at 5 days after birth (10 weeks after the last maternal dose). These were about one-third of the tolerated ustekinumab levels in pediatric patients with Crohn’s disease who received 2 mg/kg (if body weight < 40 kg) or 90 mg (if body weight ≥ 40 kg) subcutaneous ustekinumab injection [19]. The reported half-life of ustekinumab after 90 mg of subcutaneous injection was 20–31 days [20], although ustekinumab in cord blood was rapidly eliminated from the infant body. These results suggest that subcutaneous ustekinumab injection until 29 weeks of gestation may be safe for the delivered infant, as with other monoclonal antibody including anti-TNF agents [10, 13, 21–24]. Additionally, the infant in our case received routine vaccinations until the age of 6 months, and experienced no adverse events.

In conclusion, use of ustekinumab during pregnancy and lactation was feasible in this case, and the placental transfer and breast milk secretion of ustekinumab were comparable with previous reports.

## Data Availability

The datasets used and/or analyzed during the current study are available from the corresponding author on reasonable request.

## Abbreviations

UC: Ulcerative colitis
5-ASA: 5-aminosalicylate
TNF: Tumor necrosis factor
AUCs: Areas under the time-concentration curves
